# Optimization of surgical tourniquet usage to improve patient outcomes: Translational cross-disciplinary implications of a surgical practice survey

**DOI:** 10.3389/fsurg.2023.1104603

**Published:** 2023-04-17

**Authors:** Michael E. Neufeld, James A. McEwen, Julie Kerr, Arsh Sidhu, Lisa C. Howard, Bassam A. Masri

**Affiliations:** ^1^ Department of Orthopaedics, Faculty of Medicine, University of British Columbia, Vancouver, BC, Canada; ^2^ School of Biomedical Engineering, University of British Columbia, Vancouver, BC, Canada; ^3^ Western Clinical Engineering Ltd., Vancouver, BC, Canada

**Keywords:** tourniquet, optimization, outcomes, personalized, pressure, time, safety

## Abstract

Tourniquet use is common practice in many millions of orthopaedic procedures annually. Recent reviews of risks and benefits of surgical tourniquet use have primarily involved meta-analyses, many of which have forgone a comprehensive risk-benefit analysis to simply question whether “tourniquet or no tourniquet” use produces improved patient outcomes, often leading to limited, inconclusive, or conflicting results. To investigate further, a pilot survey was undertaken to determine current practices, opinions, and understandings among orthopaedic surgeons in Canada regarding use of surgical tourniquets in total knee arthroplasties (TKAs). Results of the pilot survey showed a wide range of understanding and practice associated with tourniquet use in TKAs, especially regarding tourniquet pressures and tourniquet times, two key factors known from basic research and clinical studies to impact the safety and efficacy of tourniquet use. The wide variation of use indicated by the survey results reveals important implications for surgeons, researchers, educators, and biomedical engineers, to better understand the association between key tourniquet parameters and outcomes assessed in research, which may be factors leading to their often limited, inconclusive, and conflicting results. Lastly, we provide an overview of the overly simplified assessments of tourniquet use in meta-analyses, whose conclusions may not provide an understanding of how or whether key tourniquet parameters might be optimized to retain the benefits of tourniquet use while mitigating the associated real or perceived risks.

## Introduction

Modern pneumatic surgical tourniquets consist of a microprocessor controlled pneumatic tourniquet device first invented by McEwen in 1981 to improve tourniquet safety and performance (1). This invention formed the basis of many subsequent biomedical engineering advances in pneumatic surgical tourniquet systems, leading to advances for setting personalized tourniquet pressures through the determination of an individual patient's Limb Occlusion Pressure (LOP), technology for safely and reliably auto-regulating tourniquet pressure at the set pressure level and for automatically monitoring tourniquet time, audio-visual alarms to detect and warn of clinically significant pressure variations and excessive tourniquet times, automatic calibration-checking prior to each tourniquet use, automatic detection of hazards in connected tourniquet cuffs, and other related advances related to safe tourniquet cuff design and instrumentation (2). Basic research findings and many clinical studies and observations over many years have clearly established both the benefits and the risks associated with the usage of surgical tourniquets (3–12). Benefits of using pneumatic tourniquets include establishment of a clear, dry bloodless field for improved visualization, better control of blood loss, and faster procedural completion time for improved surgical efficiency and intraoperative workflow (2, 10). Tourniquet related risks may be generally grouped into two categories: pressure-related and time-related. Pressure-related risk of injury has been shown to be related to the level of tourniquet pressure employed, and to the pressure gradients applied by specific types of tourniquet cuffs to underlying limbs (13–15). Time-related risk of injury has been shown to be related to the sustained duration of tourniquet pressurization (16). The capabilities of surgical tourniquet technology have continued to evolve to improve their safety and utility, as highlighted by the biomedical engineering advances summarized above, but the use of surgical tourniquets in clinical practice has not similarly evolved (12, 17).

In recent years reviews of the risks and benefits of surgical tourniquets have primarily involved meta-analyses, many of which have simplified the risk-benefit analysis to ask the question of whether “tourniquet or no tourniquet” use produces improved patient outcomes, often leading to limited, inconclusive, or conflicting results (18–22). Meta-analyses necessarily aggregate data and may omit, or may not be able to determine, key parameters of tourniquet usage such as: tourniquet pressure level employed and how it was determined, tourniquet pressurization time, and types of tourniquet instrumentation and cuffs used, as well as the specific association of these key parameters to patient outcomes. The overly-simplified assessments of tourniquet use in meta-analyses therefore may not provide an understanding of how or whether key tourniquet parameters might be optimized to retain the benefits of tourniquet use while mitigating the associated risks.

Tourniquet use is common practice in several million orthopaedic procedures annually in North America, thus, the ongoing pursuit of optimal tourniquet use cannot be overstated. A cross-disciplinary understanding of the impact and importance of key tourniquet parameters is essential to improve patient outcomes by incorporating the results of basic research and published clinical studies into biomedical engineering advances, with widespread learning and adoption of updated tourniquet technology and use practice. While the capabilities of surgical tourniquet technology have continued to evolve to improve their safety and utility, the lack of association between key tourniquet parameters and outcomes assessed in recent meta-analyses (18–22) demonstrate a wide range in understanding and limited, inconclusive, or conflicting risk-benefit conclusions of optimal tourniquet use.

Over the past two decades, multiple surveys have been administered in different regions and specialties assessing tourniquet practices (23–25). Results from these surveys indicate a wide variation of knowledge and application of available advances in surgical tourniquet technologies (23, 24). To investigate further, a pilot survey of surgeon members of the Canadian Arthroplasty Society (CAS) was undertaken to determine the current practices, opinions and understanding regarding of surgical tourniquet usage in total knee arthroplasties.

The survey results and resulting discussion are intended to serve as a stepping stone for future research, thereby allowing the oversimplified “tourniquet or no tourniquet” question posed by limited or inconclusive meta-analyses to be advanced to the understanding of how or whether key tourniquet parameters might be optimized to retain the benefits of tourniquet use while mitigating the associated risks and improving patient outcomes.

## Method of pilot survey

The survey was distributed to all 161 staff orthopedic surgeon members of the CAS. After review and approval by the CAS research committee the survey was distributed *via* email by CAS. The email included an online survey link and the outlined objectives of our study. The survey was administered through Microsoft Forms. The survey period was six weeks (October 2021–November 2021) and additional two follow-up emails were sent to complete the survey. The survey was anonymous and voluntary.

The survey consisted of 59 total questions that ranged from demographics to specifics on tourniquet use. The survey utilized skip logic branching to deliver the questions, as identified in the supplementary material. Non-identifiable demographic questions included: age, province of practice, job title, subspeciality training, years of practice, and type of orthopedic practice. The remaining survey questions determined if the participants were still utilizing tourniquets in their operations, and the specifics surrounding key parameters of tourniquet use. If participants were no longer utilizing tourniquets, they were redirected to an alternative pathway to complete the survey. This pathway enquired about reasons for not using tourniquets, whether they had ceased their utilization or never used tourniquets in their practice. The surgeons that indicated they currently utilize tourniquets filled the remainder of the survey which revolved around methods of tourniquet use, tourniquet time, tourniquet pressure, clinical outcomes, and tourniquet technology.

## Results of pilot survey

The results of our pilot surgical practice survey involving 91 orthopaedic surgeons (56.5% response rate) have important implications. These results are summarized in Table 1, and presented in the supplementary material. Although the preference of 90% of respondents was to use a tourniquet unless otherwise indicated, the survey revealed wide variations in knowledge of tourniquet-related basic research and clinical studies, and similarly wide variations in knowledge and application of available advances in surgical tourniquet technologies. Sixty-five percent reported that they currently use tourniquets, 25% no longer use them but previously did, and 9% never used tourniquets. For the thirty-four percent who indicated they do not currently use tourniquets in TKA procedures, the most common reasons were potential harm/risks and information provided from publications/conferences.

**Table 1:** Summary of survey results.

*Demographics*	Number of participants	Responses received	Response rate	
	161	91	56.5%	
*Tourniquet Use*	Currently use and have used in the past	No longer use but used in the past	Have never used	Currently use but did not use in the past
	59 (65%)	23 (25%)	8 (9%)	1 (1%)
*Reasons for no longer using Tourniquets*	Potential Risks and Harms	Publication/Conference Guidelines	Following standard of practice of colleagues	Perform cementless TKA
	17 (73%)	14 (61%)	3 (13%)	3 (13%)
*Understanding that reducing tourniquet pressure levels reduce the probability of tourniquet-related injuries?*	Yes	No	Not sure	
	43 (72%)	2 (3%)	15 (25%)	
*Understanding that reducing tourniquet pressure levels reduce the probability of tourniquet-related injuries?*	Yes	No	Not Sure	
	50 (83%)	2 (4%)	8 (13%)	

Per the survey's skip logic branching, the sixty-five percent of respondents who indicated they currently use tourniquets completed the remaining sections of the survey. The top reason for tourniquet use was improved visualization/bloodless field (88%), followed by performing cemented TKAs, consistency with training, and faster operative times. The most frequent adverse events reported were bruising/pinching under the tourniquet and short-term pain, which majority believed were related to improper tourniquet use (prolonged time, high-pressures, poor cuff fit), yet only 8% reported use of contoured tourniquets to better fit contoured limbs and 32% reported that they did not use limb protection beneath cuffs to minimize soft tissue injuries.

Despite substantial evidence in basic and clinical literature that patient safety and probability of harm are affected by both the duration of applied tourniquet pressurization (“tourniquet time”) and the level and gradient of applied tourniquet pressure (“tourniquet pressure”), only 83% and 72% of tourniquet users reported believing that reducing tourniquet times and pressures respectively reduces the probability of harm. Furthermore, 62% reported always using fixed pressures and 37% reported that they would modify tourniquet pressure settings based on patient parameters, most often based on systolic blood pressure and limb size, and no surgeon reported use of measuring limb occlusion pressure (LOP) to personalize the tourniquet pressure. Almost all respondents who currently use tourniquets (88%) were interested in new evidence-based guidelines regarding these parameters, a significant implication for this investigation.

## Discussion

### Variation in tourniquet practice and understanding, and its impact on meta-analyses

The wide variation in understanding of tourniquet benefits and risks and clinical tourniquet use exemplified by the survey results may be derived from the inherent limitations of the meta-analyses studying tourniquet use, specifically the heterogeneity of methods employed. Such meta-analyses necessarily aggregate data and may omit, or may not be able to determine, key parameters of tourniquet usage. Examples of key tourniquet parameters which are directly correlated to patient outcomes that should be reported directly in RCTs, and assessed for their impact in meta-analyses include:

 • The specific levels of pneumatic pressure were used, and why;
 • The specific duration of tourniquet pressurization;
 • The inflated tourniquet time relative to the overall procedure time;
 • The specific type of tourniquet cuff used for each patient, and the corresponding fit of the selected cuff to the patient's limb; and
 • The specific tourniquet instrument used (thus identifying the accuracy of pressure regulation of the device, available personalization capabilities, and safety-related information available to users).

The majority of tourniquet focused original research articles published over the past few decades do not report on the key tourniquet parameters described above. Some publications will describe the dimensions of tourniquet cuff used, and the standard protocol for setting tourniquet pressure. However, unless the research article is investigating the direct impact of a specific tourniquet parameter on the measured outcome (15), these key parameters are either omitted entirely or not provided with sufficient detail for impactful analysis.

A weakness for all meta-analyses focused on tourniquet use and impact on patient outcomes is how results from individual studies are typically compiled and grouped into “tourniquet” groups vs. “non-tourniquet” groups (18–22). Patient related outcomes are then assessed for these two groups, which typically include: the presence of serious adverse events, pain, function, quality of field of vision, blood loss, duration of surgery, survival of implant, length of hospital stay, and implant stability (18–22). Publications meeting the inclusion criteria of a meta-analysis may not have recorded tourniquet parameters, and due to the nature of aggregating meta-analysis results simply into “tourniquet” vs. “non-tourniquet” groups, the impact of key tourniquet parameters on individual outcomes is either not known or obscured when aggregated into a single “tourniquet” group. The lack of association between key tourniquet parameters and outcomes assessed in meta-analyses may lead to limited, inconclusive, or conflicting results, which cannot provide a comprehensive risk-benefit analysis of tourniquet use. Table 2 identifies the impact of key tourniquet parameters on patient outcomes, the limitation of the lack of association between tourniquet parameters and outcomes assessed in meta-analyses, and therefore the direct impact of those limitations on the risk-benefit conclusions of tourniquet use in TKA procedures. Recent level 1 evidence that tried to incorporate key parameters of optimal tourniquet use showed promising results (28).

**Table 2:** Limitations of meta-analyses for identifying key tourniquet parameters and their implications.

Key Tourniquet Parameter	Impact of Tourniquet Parameters on Patient Outcomes	Limitations of Meta-Analysis	Impact of Limitations on Meta-Analysis Conclusions
Pressure Level	High pressures can cause: - Pain; - Bruising; - Nerve damage; - PE/DVT. *Lower/personalized pressure levels reduce risk of negative outcomes*.	No association between - Pressure levels reported in each study or patient; and - Impact of pressure levels on the reported outcomes (e.g., pain, adverse events).	- Frequency/severity of negative outcomes are proportionate to level of pressure utilized. - Outcome reporting should be assessed per similar applied pressure groups. - Generic “tourniquet group” assessments provide misleading results due to variability of tourniquet pressures utilized.
Pressurization time	Long pressurization times can cause: - Pain; - Bruising; - Nerve damage; - PE/DVT. *Shorter pressurization times reduce risk of negative outcomes*.	No association between - Pressurization time for each patient; and - Impact on the reported outcomes. Note: Individual RCTs may not record mean, minimum, or maximum tourniquet pressurization times employed in the study, and may not compare tourniquet pressurization times to overall procedure times	- Frequency/severity of negative outcomes are proportionate to total pressurization time. - Outcome reporting should be assessed per similar total pressurization times. - Generic “tourniquet group” assessments provide misleading results due to variability of tourniquet pressurization times employed. - Not reporting individual pressurization times relative to outcomes may obscure potential benefits of tourniquet time optimization
Tourniquet cuff type	Cuff Shapes - Cylindrical vs. Contour; - Wide vs. Narrow. *Wider cuffs allow lower and safer personalized pressures to be used*.	No reporting, association, or analysis on the cuff technology utilized in the included studies. Note: Individual RCTs may not record the specific tourniquet cuffs utilized in the study.	- Negative outcomes in generic “tourniquet groups” may be influenced by the type of tourniquet cuff technology used in a specific study or on an individual patient.
Fit of cuff on limb	A poor fitting cuff may be: - Too loose, requiring higher pressures to effectively occlude arterial blood flow; - Too tight, leading to venous congestion; - Not matched to limb shape *Cuffs matched to limb shapes allow use of lower and safer pressure levels and pressure gradients*.	No reporting, association, or analysis on the assessment of fit of cuff on a given patient's limb. Note: Individual RCT's typically do not record the assessment of fit of the cuff on a limb.	- Frequency/severity of negative outcomes are proportionate to the quality of the fit of a cuff on a patient's limb. - Poor fitting cuffs may require higher pressures to occlude blood flow, which increases risk of use. - Generic “tourniquet group” assessments provide misleading results due to inadequate analysis of cuff fit and its impact on tourniquet pressures and pressure gradients.

Our pilot survey results indicate a wide range of understanding and practice associated with tourniquet use in TKA procedures. This variation of tourniquet use is likely to correspond to a variation in both tourniquet efficacy and patient outcomes. A single “tourniquet use group” which includes variation of key tourniquet parameters across the included studies of a meta-analysis therefore provides a skewed representation of results. For example, the Cochrane systematic review and meta-analysis by Ahmed et al. identifies a range of tourniquet pressures from the included studies; pressures based upon SBP plus an added margin, twice the systolic blood pressure, 250 mmHg, 275 mmHg, 300–350 mmHg, 360–380 mmHg, 400 mmHg, 225 or 300 mmHg (surgeon preference), 13.3 kPa (100 mmHg), and 0.8 bar (600 mmHg) (18). Mean tourniquet times for the included studies were not identified as part of the review. As the review only assesses outcomes for the general tourniquet group, it is unclear how the various pressures are correlated to the outcome measures. In order to derive any accurate and valuable conclusions from such meta-analysis, the outcomes should be further assessed by the specific parameters of tourniquet usage.

### Optimizing tourniquet pressures

From a cross-disciplinary perspective, recent advances in biomedical engineering have led to advances in technology which can be adapted to the needs of individual patients, surgeons and procedures. One key advancement in technology is the ability to personalize the tourniquet pressure such that lower, effective pressures are employed (17, 26). Other advances in surgical tourniquet technology include instruments capable of very accurate regulation of set pressure; improved tourniquet cuffs capable of producing safer low pressure gradients on underlying patient limbs (29); improved tourniquet systems capable of automatically applying pressure for the shortest time periods deemed necessary by surgeons during specific procedures, and advanced tourniquet systems which can be adapted to the needs of individual patients, surgeons and procedures (17).

Basic research and clinical studies have clearly demonstrated that higher tourniquet pressures are associated with higher risks of tourniquet-related injuries (1, 7, 8, 14, 30). One of the key advances in available technology include improved types of tourniquet instruments capable of automatic determination Limb Occlusion Pressure (LOP), to inform the lowest and safest effective pressure level for individual patients (17, 29, 30). LOP is defined as the minimum pressure required, at a specific time in a specific type of tourniquet cuff applied to a specific patient's limb at a specific location, to stop the flow of arterial blood into the limb distal to the cuffs (2, 17, 30). Once LOP has been determined for a patient, tourniquet pressure is set by adding a safety margin to the LOP to account for physiologic changes and intraoperative variations (17, 30).

Personalizing tourniquet pressures based upon a patient's LOP has been verified and validated to ensure an effective bloodless surgical field, while maintaining personalization of the applied tourniquet for each patient (11, 17, 29). Personalized tourniquet pressure based upon LOP is typically substantially lower than standard tourniquet pressures currently used, which can reduce the risks of tourniquet-related injuries (2, 15, 30–32). Survey results showed 37% of tourniquet users make efforts to modify standard tourniquet pressure settings based on patient parameters such as systolic blood pressure and limb size, although both parameters have been shown to be inferior to settings based on LOP (26, 27). Lower pressures are also associated with lower pressure gradients, which further reduces risk of nerve damage (13, 14). In addition, the type of tourniquet cuff selected and its fit on the patient's limb can impact the efficacy of the applied pressure and the resultant pressure gradients as lower limbs vary in both size and shape; some upper thighs are cylindrical, while others are more tapered. A wide cuff should be selected, in either a contour or cylindrical style, to best fit the specific patient's limb (29). A properly fitting cuff will result in lower required pressures, with resulting reduced pressure gradients, which will decrease the risk of adverse events (13, 29).

Despite the evidence which demonstrates the use of LOP substantially reduces tourniquet pressures while being safe and effective, no respondent in the survey indicated that they set tourniquet pressure based on a patients LOP. Because basic research and clinical studies have shown the impact of lower tourniquet pressures and pressure gradients, and because biomedical engineering advances now allow this to be done safely, efficiently, and effectively in practice, investigation of the impact on outcomes with a view to optimizing tourniquet use may be timely.

### Optimizing tourniquet pressurization times

Basic research and clinical studies have clearly demonstrated that shorter applied tourniquet pressurization times are associated with lower risks of tourniquet-related injuries (5, 16, 19, 33–35). Personalizing applied tourniquet pressurization time to both patient, procedure, and surgeon, without sacrificing the benefits of tourniquet use, may significantly improve patient outcomes. Tourniquet pressurization time is largely dependent on procedure complexity, surgeon skill, and surgeon preferences. This can lead to wide variations in applied tourniquet pressurization times. When asked to report average tourniquet pressurization times, 70% of tourniquet users indicated times for primary TKA were in the 30–60-minute range, and twice that time for a revision TKA. These times are significant, particularly when the corresponding pressures are non-personalized, fixed pressures which may be significantly higher than required, based on the results of the pilot survey.

Minimizing applied tourniquet pressurization time reduces the risk of tourniquet-related injuries (16), however it is currently unclear how to optimize the timing such that both patient and surgeon maximize the benefits of tourniquet use while minimizing risk. There are limited studies on the efficacy of tourniquet use and cemented implants, particularly due to the long follow-up time required for relevant results (36). For a straightforward primary TKA, it may be possible to significantly reduce tourniquet pressurization time by only inflating the tourniquet for aspects of a procedure where a clear, dry surgical field are critical, and deflating the tourniquet when the benefit is less critical. However, a revision TKA often provides unique challenges for each patient, and therefore tourniquet pressurization time is longer and its use is more valued. Investigating specific aspects of both primary and revision TKAs for which the tourniquet can be inflated as well as deflated, by making use of available biomedical engineering advances, may be useful for optimizing tourniquet use by minimizing applied tourniquet pressurization times to improve patient outcomes.

## Conclusion

Meta-analyses focused on the sole question of “tourniquet or no tourniquet” use have led to limited, inconclusive, and conflicting results and conclusions. Future investigations may be better framed and advanced to ask more comprehensive questions about how or whether key tourniquet parameters might be optimized to retain the benefits of tourniquet use while mitigating the associated risks. Our survey demonstrated that there is a wide range of understanding and practice associated with tourniquet use in TKAs, and that while there is such variation, a general conclusion regarding the safety and efficacy of tourniquet use in TKA procedures is impractical. In order to advance this discussion, researchers of pneumatic surgical tourniquet use should include specific details regarding key tourniquet parameters when describing both methods and limitations of a study. Correspondingly, when meta-analyses of tourniquet use are published, they should include a comprehensive risk-benefit analysis of the association between key tourniquet parameters and patient outcomes, or at minimum clearly state that their conclusions are limited by the omission of key parameters.

Our survey and associated discussions identify considerable variability in tourniquet use with respect to several key parameters, as well as a need for further research and updated guidelines regarding key parameters of safe tourniquet usage to optimize tourniquet use in Orthopaedics. We have outlined the mechanisms for operationalizing the results of basic science research and clinical studies by translating advances in biomedical engineering and adapting the characteristics of surgical tourniquets so that they can be readily employed in clinical practice. The individual key tourniquet parameters can and should be studied in order to optimize the use of tourniquets with a goal to retain the benefits and improve patient outcomes.

## Data Availability

The original contributions presented in the study are included in the article/Supplementary Material, further inquiries can be directed to the corresponding author.
