# Supplementary material for: Optimization of surgical tourniquet usage to improve patient outcomes: Translational cross-disciplinary implications of a surgical practice survey
Source: Front Surg. 2023 Apr 17;10:1104603. doi: 10.3389/fsurg.2023.1104603 (PMC10149658; doi:10.3389/fsurg.2023.1104603)
Supplement: Supplementary file 1 [file Table1.pdf]

Tourniquet Use in Total Knee Arthroplasty: A Survey of Current Practice and Preferences of Canadian Orthopaedic Surgeons - Formatted questions with associated follow-ups

**Section 1 - Demographics**
1. What is your age?<br><30<br>30-39<br>40-49<br>50-59<br>>60
2. What Province do you currently practice in?<br>Alberta<br>British Columbia<br>Manitoba<br>New Brunswick<br>Newfoundland and Labrador<br>Northwest Territories<br>Nova Scotia<br>Nunavut<br>Ontario<br>Prince Edward Island<br>Quebec<br>Saskatchewan<br>Yukon
3. What best describes your position?<br>Staff Surgeon/Attending<br>Clinical Fellow<br>Locum physician
4. Did you complete formal subspecialty fellowship training in hip and knee arthroplasty?<br>Yes<br>No

5. Including fellowship(s), how many years of practice do you have as an orthopaedic surgeon?

> 5 years
5 - 10 years
11 - 20 years
> 20 years

6. What type of orthopaedic practice do you work in?

Academic Centre
Community Centre
Rural
Private Practice
Other? _____________

7. How many TKA procedures do you perform a year?

<10
10-25
26-50
51-100
>100

8. What percentage of your practice does arthroplasty make up?

<25
25-50
51-75
>75

9. Do you prefer cemented or cementless TKA?

Cemented
Cementless
Depends on the patient
Other: __________

10. What is your history with tourniquet use in TKA?<br>a. Currently use and have used in the past<br>b. Currently use but did not use in the past<br>c. No longer use but used in the past<br>d. Have never used	If answered a, go to Section 2<br>If answered b, go to Q11<br>If answered c, go to Q12<br>If answered d, go to Q15

11. Why do you now use tourniquet when you did not in the past? (Check any that apply)<br>Used in training<br>Perform cemented procedure<br>Improved visualization with a bloodless field<br>Less blood loss<br>Faster operative time<br>Believe if used correctly that outcomes and harms similar to no tourniquet<br>Other: _______________	Go to Section 2
12. Why do you no longer use tourniquets when you did in the past? (Check any that apply)<br>Publication / conference guidelines<br>Potential risks / harms<br>Following standard of practice of groups / colleagues<br>Now perform cementless TKAs (bloodless field not required)<br>Other: __________	Go to Q13
13. Do you think that tourniquet use negatively affects clinical outcomes in terms of pain, function, patient reported outcomes?<br>Yes – short term effects<br>Yes – long term effects<br>Only if used incorrectly<br>Undecided<br>Other: _________	Go to Q14
14. Do you think that tourniquet use is associated with an increased risk of complications?<br>Yes<br>No<br>Undecided<br>Only if used incorrectly	Go to Section 7 Q59 and end survey

15. Why have you never used tourniquets in your practice? (Short answer)	Go to Q16
16. Do you have any other comments on tourniquet use? (Short answer)	End survey
**Section 2 – Tourniquet Use**	
17. Do you use tourniquets in:<br>Primary TKA only<br>Revision TKA only<br>Both Primary and Revision TKA	
18. During your training were tourniquets routinely used for for TKA during training?<br>Yes<br>No<br>Selective usage	
19. What percentage of TKA procedures would you use a tourniquet for?<br>< 25%<br>25% - 50%<br>51% - 75%<br>76% - 99%<br>All procedures (100%)	
20. What is your current preference regarding tourniquet use?<br>To use a tourniquet, unless otherwise indicated<br>To not use a tourniquet, unless otherwise indicated	
21. What patient factors influence tourniquet use? (Check any that apply)<br>Peripheral vascular disease<br>BMI<br>Diabetes<br>Sex<br>Other contraindications<br>Other: _______	

22. What surgical factors influence tourniquet use? (Check any that apply)
    Anticipated OR time
    Cemented vs Cementless
    Primary vs Revision
    Surgical Approach
    None of these
    Other: __________

23. What are the reasons for using a tourniquet in surgery? (Check any that apply)
    Improved visualization with a bloodless field
    Used in training
    Perform cemented procedure
    Faster operative time
    Less blood loss
    Believe if used correctly that outcomes are better than not using a tourniquet
    Other: ______________

24. What are the reasons for not using a tourniquet in surgery? (Check any that apply)
    Not used in training
    Perform cementless procedure
    Risks of tourniquet use do not outweigh the benefit
    Do not require tourniquet for a bloodless field for visualization in modern TKA
    Believe tourniquets lead to more pain and poorer outcomes
    Insignificant difference in blood loss
    Tourniquet cuff cannot be applied proximal to surgical location
    Other: __________________

Section 3 – Tourniquet Time

25. Is it your understanding that reducing tourniquet time reduces the probability of tourniquet-related injuries?
    Yes
    No
    I'm not sure

26. What is your average tourniquet time for primary TKA?

< 30 minutes
31 - 60 minutes
61 - 90 minutes
91 - 120 minutes
> 120 minutes
Other: _______

27. What is your average tourniquet time for revision TKA?

< 30 minutes
31 - 60 minutes
61 - 90 minutes
91 - 120 minutes
> 120 minutes
Other: _______

28. What is the minimum tourniquet time you employ?

< 15 minutes
30 minutes
45 minutes
60 minutes
90 minutes
120 minutes

29. What is the maximum tourniquet time you employ?

15 minutes
30 minutes
45 minutes
60 minutes
90 minutes
120 minutes
180 minutes
240 minutes

30. At what elapsed tourniquet time would you deflate the tourniquet cuff to allow for reperfusion and then reinflating?
 60 minutes
 90 minutes
 120 minutes
 180 minutes
 240 minutes
 I do not typically require reperfusion time
 Other: ____________________

31. When do you inflate the tourniquet?
 Start of the procedure
 At the time a bloodless field is required
 The cuff will be applied to the patient, but the tourniquet remains deflated unless required to stop bleeding
 Other: ______________________

32. When do you deflate the tourniquet?
 At the completion of the procedure
 Whenever a bloodless field is not required
 Prior to wound closure to allow for hemostasis
 Other: ______________________

33. How do you typically deflate the tourniquet?
 Rapid deflation
 Cyclic deflation
 Stepped deflation
 Other: _____________________

34. Are you interested in a new evidence-based guideline for reducing tourniquet time?
 Yes
 No

Section 4 – Tourniquet Pressure

35. Is it your understanding that reducing tourniquet pressure levels reduce the probability of tourniquet-related injuries?
 Yes
 No
 I'm not sure

36. What kind of limb protection do you typically apply under the tourniquet to protect the skin?<br>Cast padding / Webril<br>Stockinette<br>Manufacturer supplied limb protection sleeve<br>None	
37. What patient factors are important to you when deciding how to set tourniquet pressure? (Check any that apply)<br>Patient age<br>Limb size/shape<br>Blood Pressure<br>Fit of the tourniquet cuff on the operative limb<br>Other: ____________________	
38. How do you currently set tourniquet pressure?<br>a. Fixed value<br>b. Personalized – Limb Occlusion Pressure (LOP) measurement<br>c. Based upon SBP<br>d. Other: ______________________	If answered a, go to Q40<br>If answered b, go to Q42<br>If answered c, go to Q44<br>If answered d, go to Q39
39. Please explain your decision process for setting a tourniquet pressure. (Short answer)	Go to Q45
40. What fixed pressures do you typically select? (Select multiple if appropriate)<br>150 mmHg<br>200 mmHg<br>250 mmHg<br>300 mmHg<br>350 mmHg<br>Other: _________________	Go to Q41
41. Will you modify your fixed pressure based upon the patient's limb size?<br>Yes<br>No<br>Other: _________________	Go to Q45
42. How do you measure Limb Occlusion Pressure (LOP)?<br>Manually with a Doppler ultrasound probe<br>Automatically using a photoplethysmography sensor (distal sensor)<br>Other:______________________	Go to Q43

43. What margin of safety do you add to the LOP measurement to set the tourniquet pressure?<br>LOP based – Use the Recommended Tourniquet Pressure (RTP) automatically recommended by the tourniquet instrument<br>LOP based (smaller margin for low LOP, larger margin for high LOP)<br>Fixed margin of 50 mmHg<br>Fixed margin of 100 mmHg<br>Other: __________________	Go to Q46
44. What margin do you add to SBP to set tourniquet pressure?<br>25 mmHg<br>50 mmHg<br>75 mmHg<br>100 Hg<br>Other: ____________	Go to Q45
45. Would you be interested in adapting your tourniquet use protocol to use personalized pressures based upon LOP?<br>Yes – I am interested in updating our clinical protocols for improved patient outcomes by personalizing tourniquet device use<br>Maybe – I am interesting in reading evidence-based guidelines for personalized tourniquet device use<br>No – The tourniquet protocol I follow provides acceptable surgical outcomes	Go to Q46
46. Would you be interested in using new technology developed to allow for simple, effective, and reliable LOP measurements to personalize tourniquet use?<br>Yes<br>No<br>Maybe	Go to Section 5
**Section 5 – Clinical outcomes**	
47. Do you think that tourniquet use negatively effects clinical outcomes in terms of pain, function, patient reported outcomes?<br>No<br>Only if used incorrectly<br>Undecided<br>Yes	

48. Do you think that tourniquet use is associated with an increased risk of complications?
  - No
  - Only if used incorrectly
  - Undecided
  - Yes

49. Have you ever experienced adverse outcomes potentially associated with tourniquet use? Check any that apply.
  - None
  - Bruising/pinching under cuff
  - Venous Thromboembolism (DVT/PE)
  - Infection
  - Patient’s complaint of pain
  - Longer recovery time
  - Other: ______________________

50. Are you aware of any reports from colleagues about adverse outcomes potentially associated with tourniquet use? Check any that apply.
  - None
  - Bruising/pinching under cuff
  - Venous Thromboembolism (DVT/PE)
  - Infection
  - Patient’s complaint of pain
  - Longer recovery time
  - Other: ______________________

51. Why do you think these outcomes (if any) occurred? (Check any that apply)
  - Not Applicable
  - High tourniquet pressure
  - Long tourniquet time
  - Patient history
  - Poor fitting cuff
  - Chemicals under cuff
  - Lack of limb protection
  - Other: _______________

Section 6 – Tourniquet Technology and available resources

52. What type of tourniquet cuff do you typically prefer to use?<br>a. Single use (sterile)<br>b. Reusable	If answered a, go to Q53<br>If answered b, go to Q54
53. Why do use single use tourniquet cuffs?<br>Hospital procurement decision<br>Require the sterile feature of the cuff<br>Other: ______________	Go to Q55
54. Why do you use reusable tourniquet cuffs?<br>Hospital procurement decision<br>No need for sterile use<br>Other: ______________	Go to Q55
55. What shape of tourniquet cuff do you prefer to typically use?<br>a. Cylindrical<br>b. Contour	If answered a, go to Q56<br>If answered b, go to Q57
56. Why do you use cylindrical cuffs?<br>Hospital procurement decision<br>Patient limb shape<br>Other: ______________	Go to Q58
57. Why do you use contour cuffs?<br>Hospital procurement decision<br>Patient limb shape<br>Other: ______________	Go to Q58
58. What are the limitations to using tourniquets in a personalized way? (Personalized pressures, reduced inflation time, use of contour cuffs)<br>Inventory<br>Time<br>Funding / cost<br>Other: ______________	
**Section 7 – Final comments**	
59. Are there any additional details you wish to include regarding your current tourniquet use practice?	
