# Supplementary material for: Optimization of surgical tourniquet usage to improve patient outcomes: Translational cross-disciplinary implications of a surgical practice survey
Source: Front Surg. 2023 Apr 17;10:1104603. doi: 10.3389/fsurg.2023.1104603 (PMC10149658; doi:10.3389/fsurg.2023.1104603)
Supplement: Supplementary file 2 [file Presentation1.pdf]

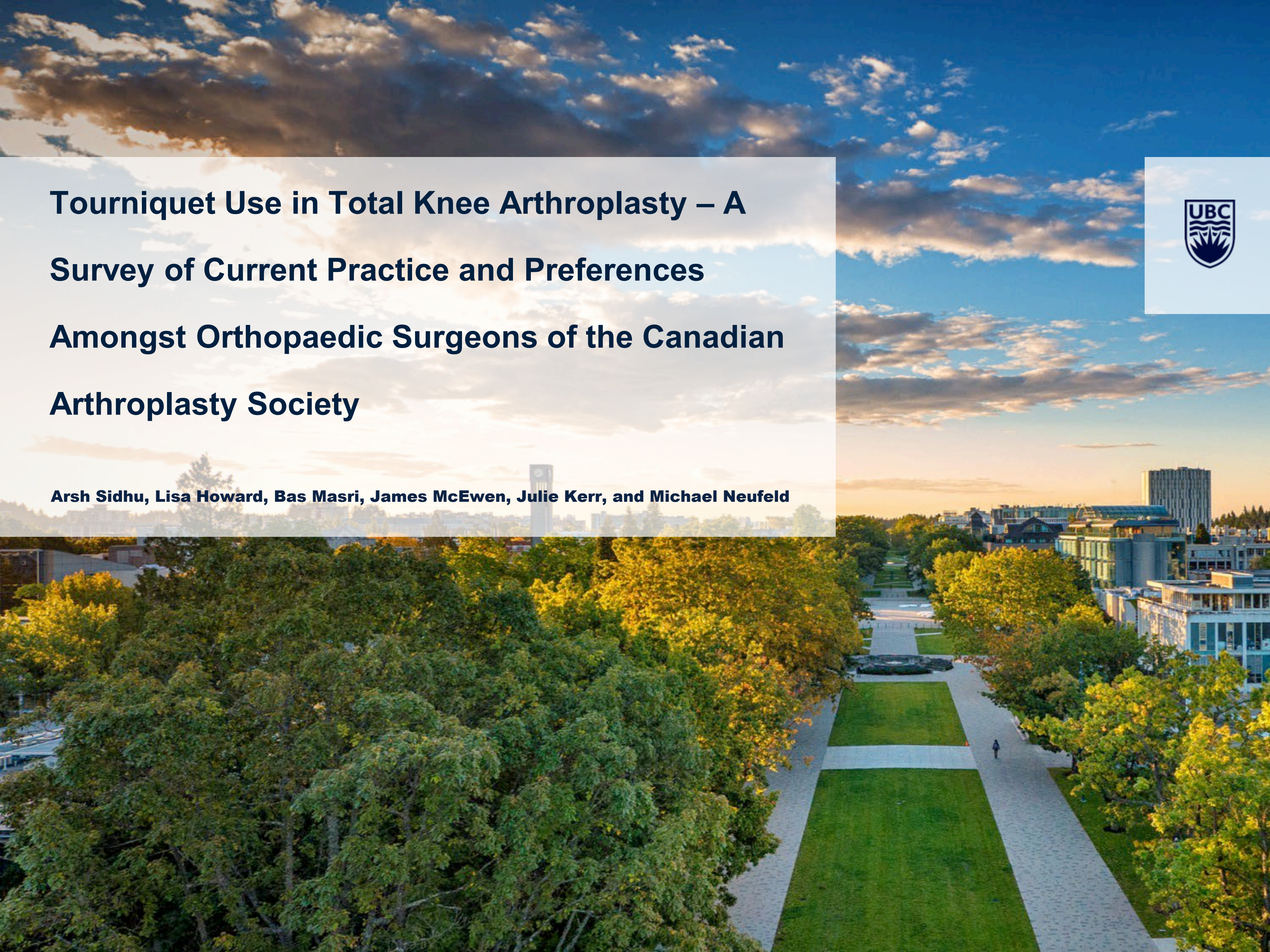

Tourniquet Use in Total Knee Arthroplasty – A Survey of Current Practice and Preferences Amongst Orthopaedic Surgeons of the Canadian Arthroplasty Society

Arsh Sidhu, Lisa Howard, Bas Masri, James McEwen, Julie Kerr, and Michael Neufeld

Disclosures

- **Nothing to disclose**

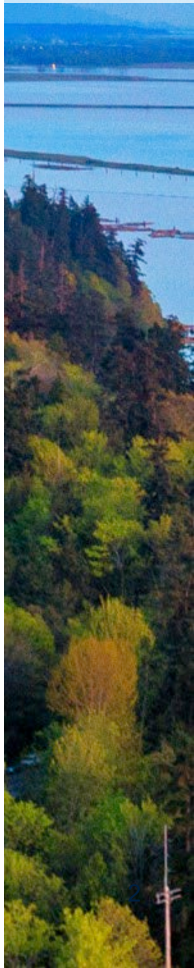

Acknowledgements

- UBC Orthopaedics Department of Lower Limb Reconstruction and Oncology
- James McEwen, PhD
- Julie Kerr
- Lisa Howard, MD
- Bas Masri, MD
- Michael Neufeld, MD

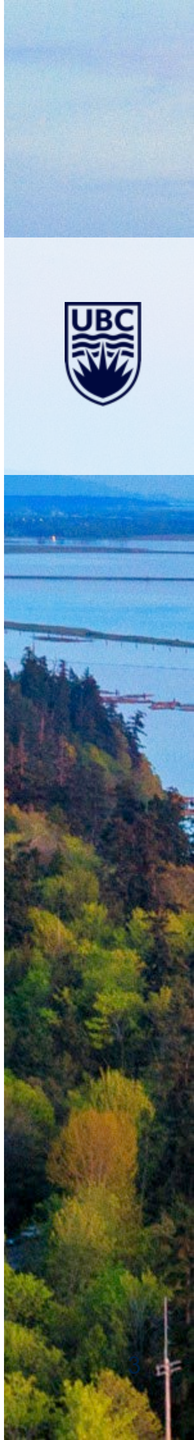

Background

- Tourniquets during knee arthroplasty historically used for:
  - Decrease blood loss
  - Improve intraoperative visibility
  - Assist in better cement digitation

- Recent literature has questioned the safety of tourniquets in TKA

- Tourniquet use in the USA = 95%

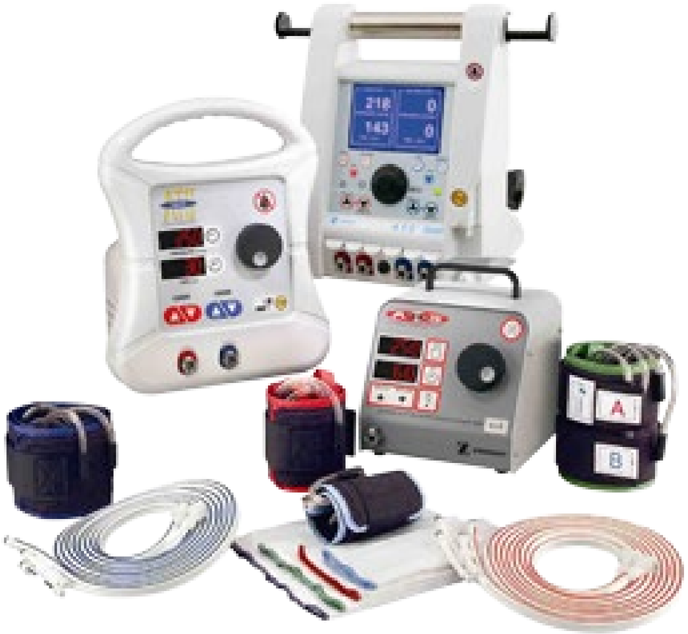

Purpose

- Determine Tourniquet use practices and preferences among surgeons in Canadian Arthroplasty Society for TKA surgery
- Need and interest for national guidelines regarding best clinical practice for tourniquet use in TKA
- Identify areas that require further study in tourniquet use

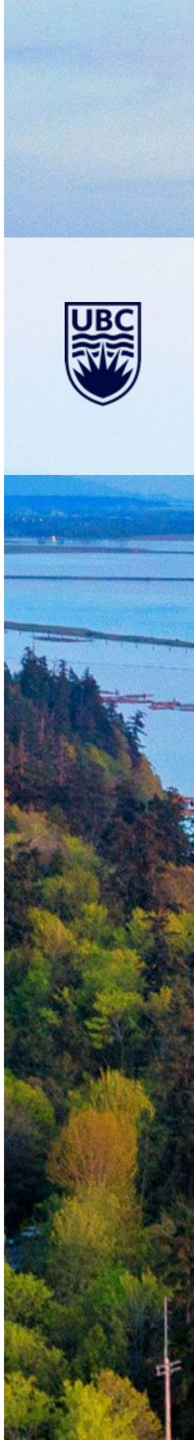

Methods

- Self- administered survey to members of the Canadian Arthroplasty Society (CAS)

- Delivered over 6 weeks to a total of 161 participants
- Skip Logic branching for maximum 59 questions

Canadian Arthroplasty Society
Société canadienne d'arthroplastie

Methods

- Demographics
- Tourniquet
    - Use
    - Parameters
    - Rationale for and against

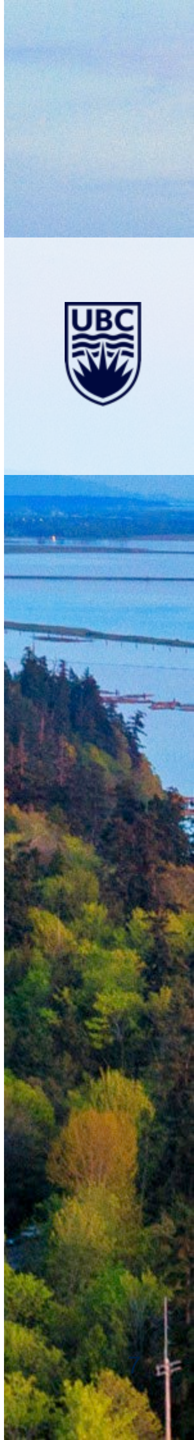

Results

- 57 % response rate (91/161 respondents)
- Demographics
  - All Staff surgeons
  - 88% fellowship trained in hip and knee arthroplasty
  - 59 % worked in an academic setting
  - 74% completed >100 TKA in a year
  - 65% preferred cemented TKA

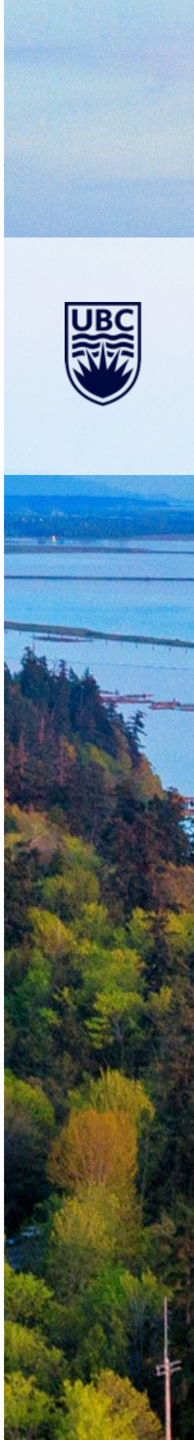

Results

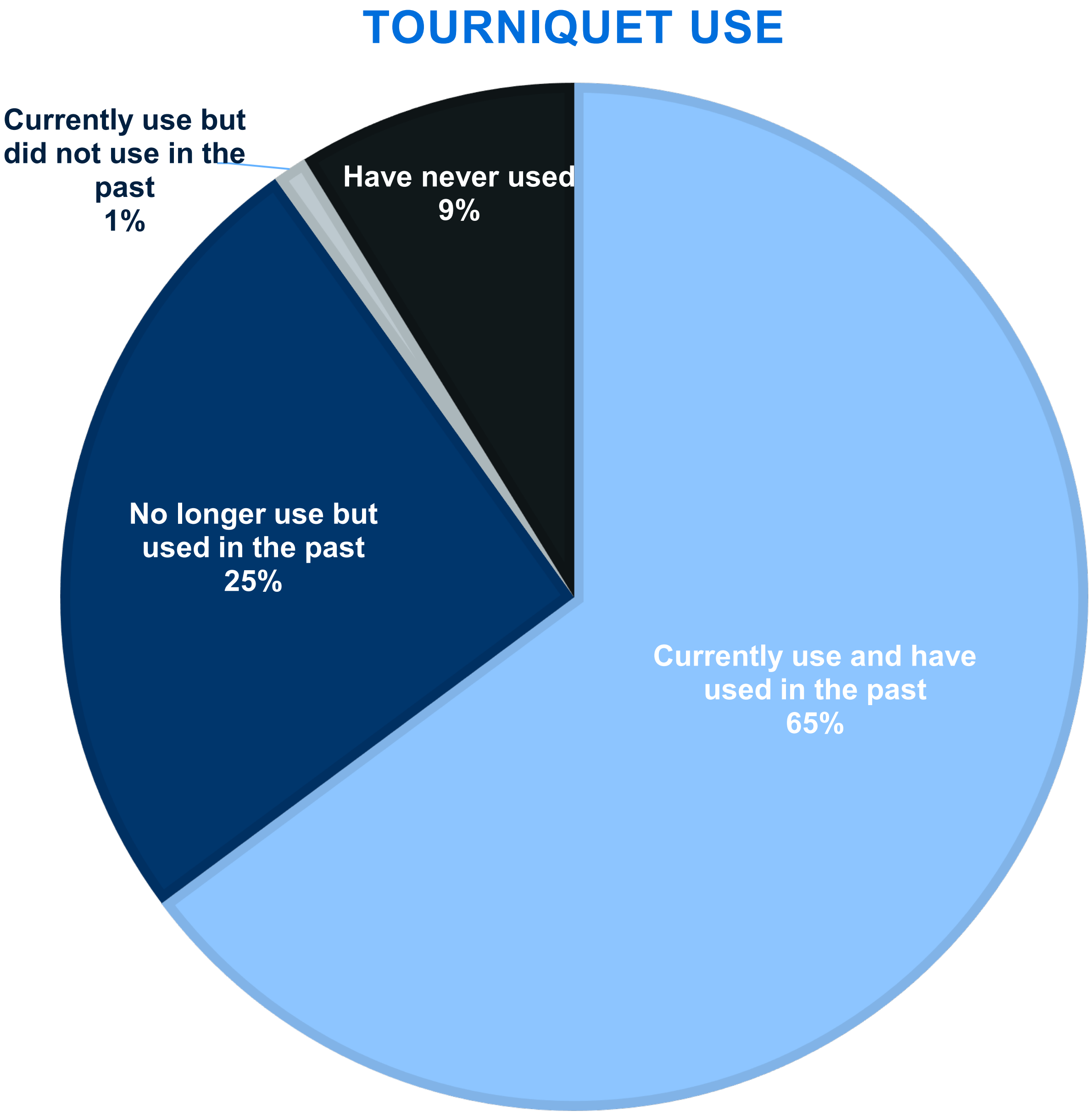
TOURNIQUET USE
Currently use but did not use in the past 1%
Have never used 9%
No longer use but used in the past 25%
Currently use and have used in the past 65%

Previous Users

Reasons for not using:

- 74% potential risks/harms
- 61 % publication /conference guidelines
- 13% following standard practice of colleagues
- 13% perform cementless TKA

Results – Tourniquet Users

- 97% used tourniquets in over 50% of their cases
- 90% preferred to use tourniquets unless otherwise indicated
- Patient Factors influencing use:
  - PVD (85%)
  - BMI

- Surgical Factors:
  - Anticipated OR time
  - Cemented vs Cementless

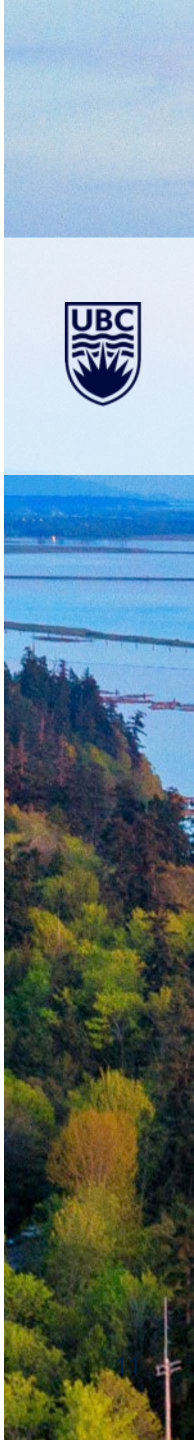

Results – Tourniquet Users

- Reasons for use:
  - 88 % believed it improved visualization with a bloodless field
  - 66% cemented procedures
  - 42% because utilized tourniquets in training

Results – Tourniquet Users

- Perception of tourniquets:
  - 45% believed association with negative short term outcomes of pain, function, or patient reported outcomes

- Perception for adverse events reasons
  - 50.0% long tourniquet times
  - 43.3% high tourniquet pressure
  - 30% poor fitting cuff

Results – Tourniquet time Primary TKA

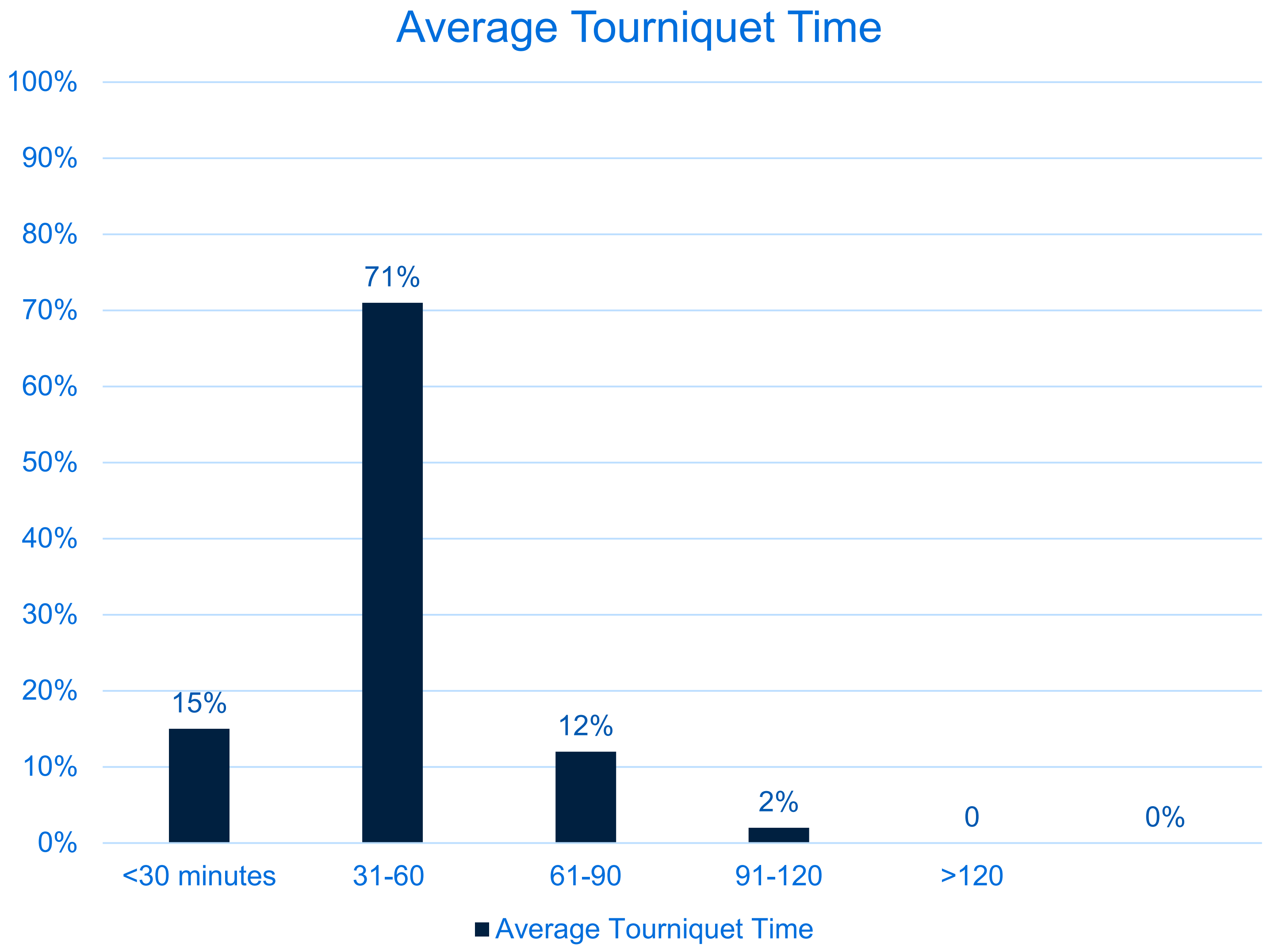

Results – Tourniquet time

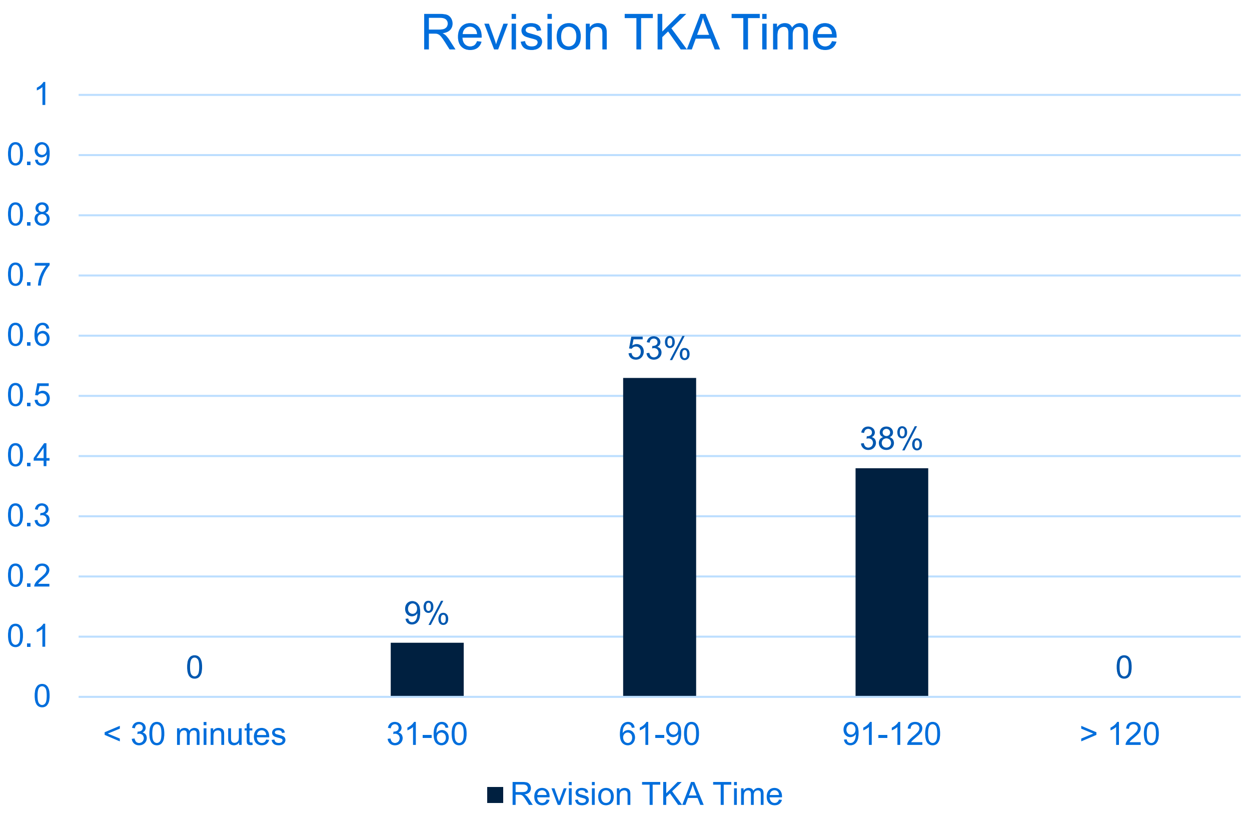

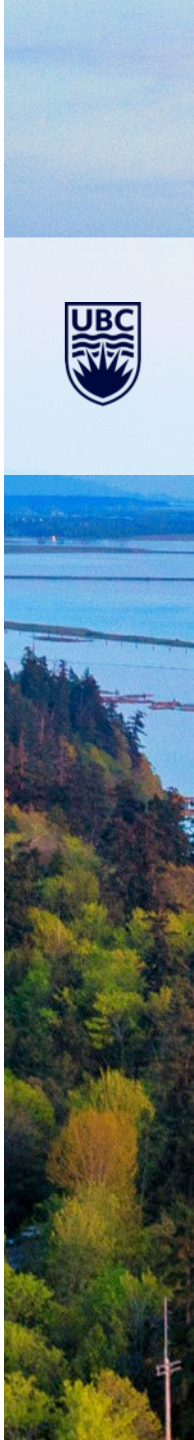

Results – Tourniquet time

- Variable Maximum tourniquet time – 55% reported 120 minutes
- Tourniquet inflation:
    - 85% inflated at start of the procedure
    - 50% deflated tourniquet prior to wound closure

Results – Tourniquet pressure

- 72% believed reduced tourniquet pressure reduced probability of tourniquet related injuries
- Important patient factors in setting tourniquet pressure
  - Blood Pressure – 78%
  - Limb Size – 59%
  - Fit of the tourniquet

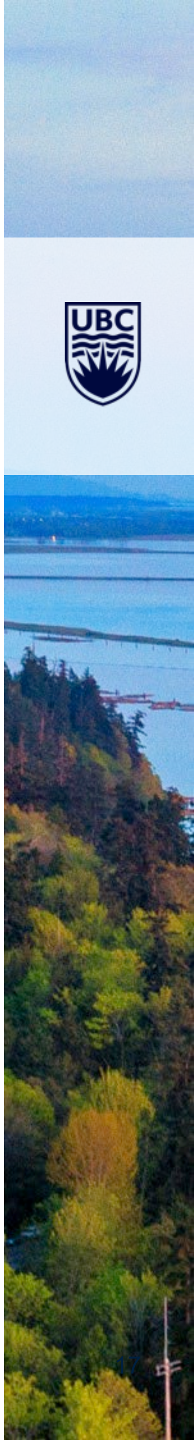

Results – Tourniquet pressure

Tourniquet Pressures:

- Fixed value (60%)
- Based upon SBP (40%)

- No one utilized personalized pressures in the form of LOP

Results – Tourniquet Types/Protection

- Protection
  - 32% used no protection
  - 68% used webril or stockinette

- Type: Used indicated by hospital decision
  - Over 90% used cylindrical reusable cuffs

Results – Tourniquet Types/Protection

- 88% indicated interest in new evidence-based guideline for reducing tourniquet time
- 86% interested in evidence based guideline for reducing pressure with personalized pressure

Limitations

- CAS Surgeons Surveyed
- 57% response rate

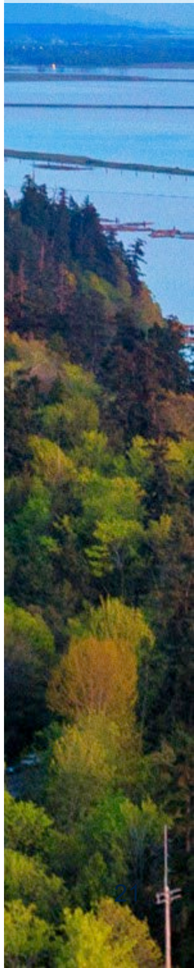

Discussion

- Current usage of tourniquets still prominent at 65%

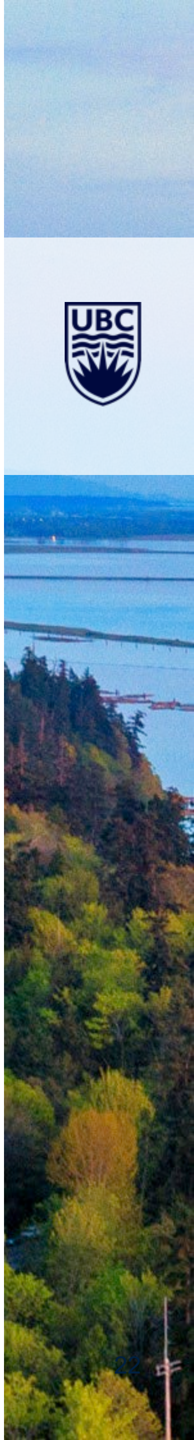

Discussion

Parameters important role in reducing tourniquet harm and optimizing use

- Pressure
- Time
- Fit

Variability of tourniquet use persists

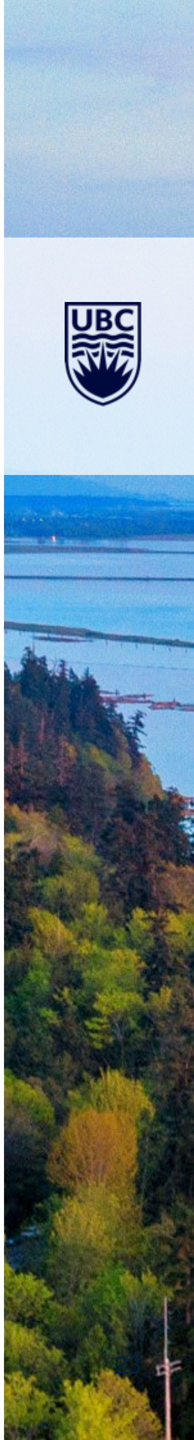

Conclusion

- Tourniquet use in TKA remains an important topic among arthroplasty surgeons in the CAS

- Considerable interest and need for further research and updated guidelines regarding key parameters of safe tourniquet usage to optimize tourniquet use in TKA.

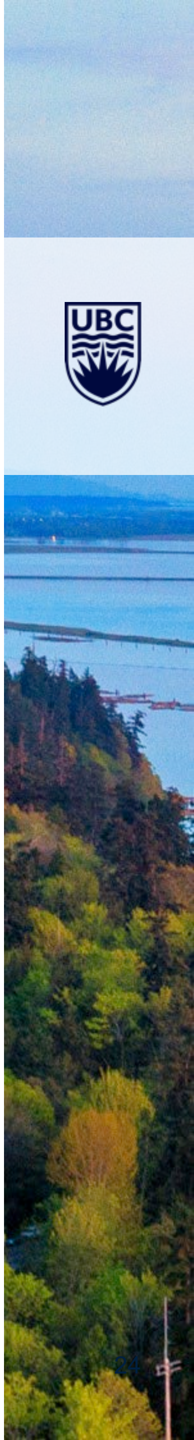

Conclusion

Thank You

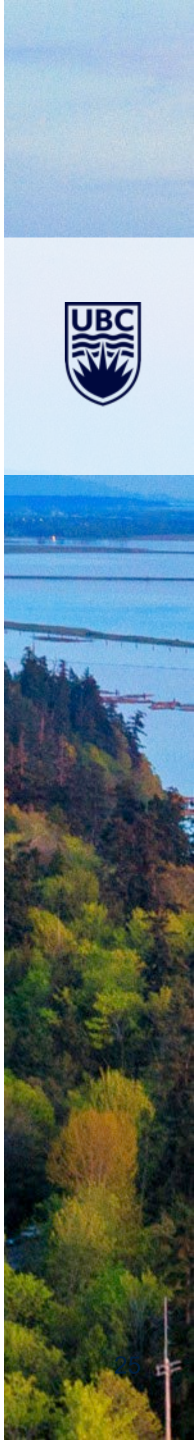

Results – Years of Practice

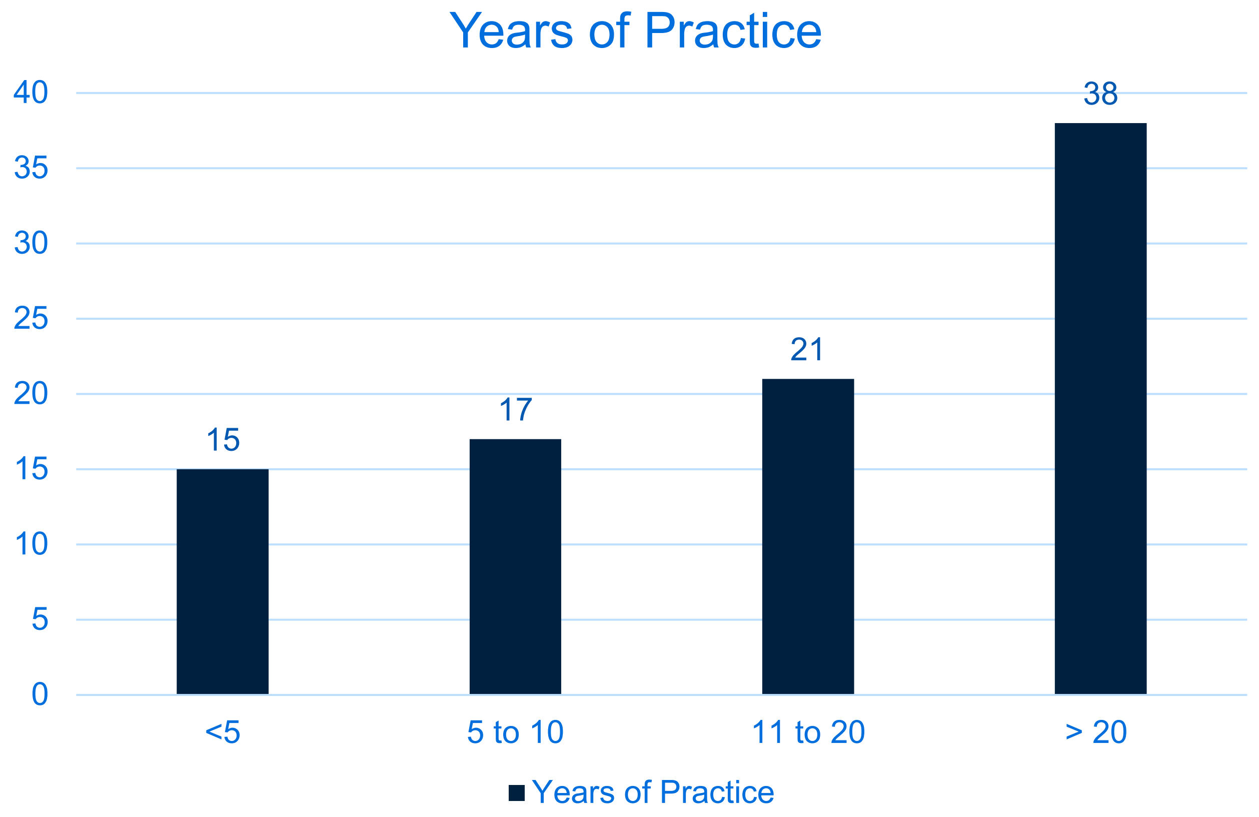

- 55% greater than 10 years of practice

Provincial Breakdown

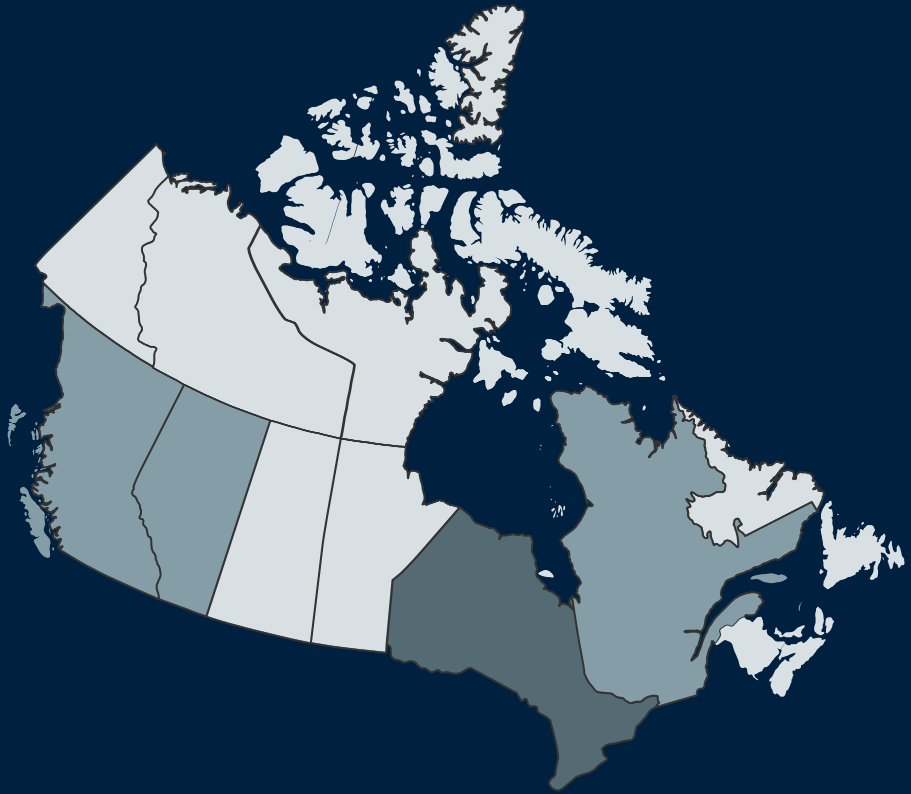

1.	Ontario	40
2.	British Columbia	12
3.	Alberta	10
4.	Nova Scotia	9
5.	Quebec	8
6.	Manitoba	5
7.	Saskatchewan	4
8.	New Brunswick and Newfoundland	3
9.	Prince Edward Island	0
10.	Yukon, Nunavut, NWT	0

Results – Tourniquet Users

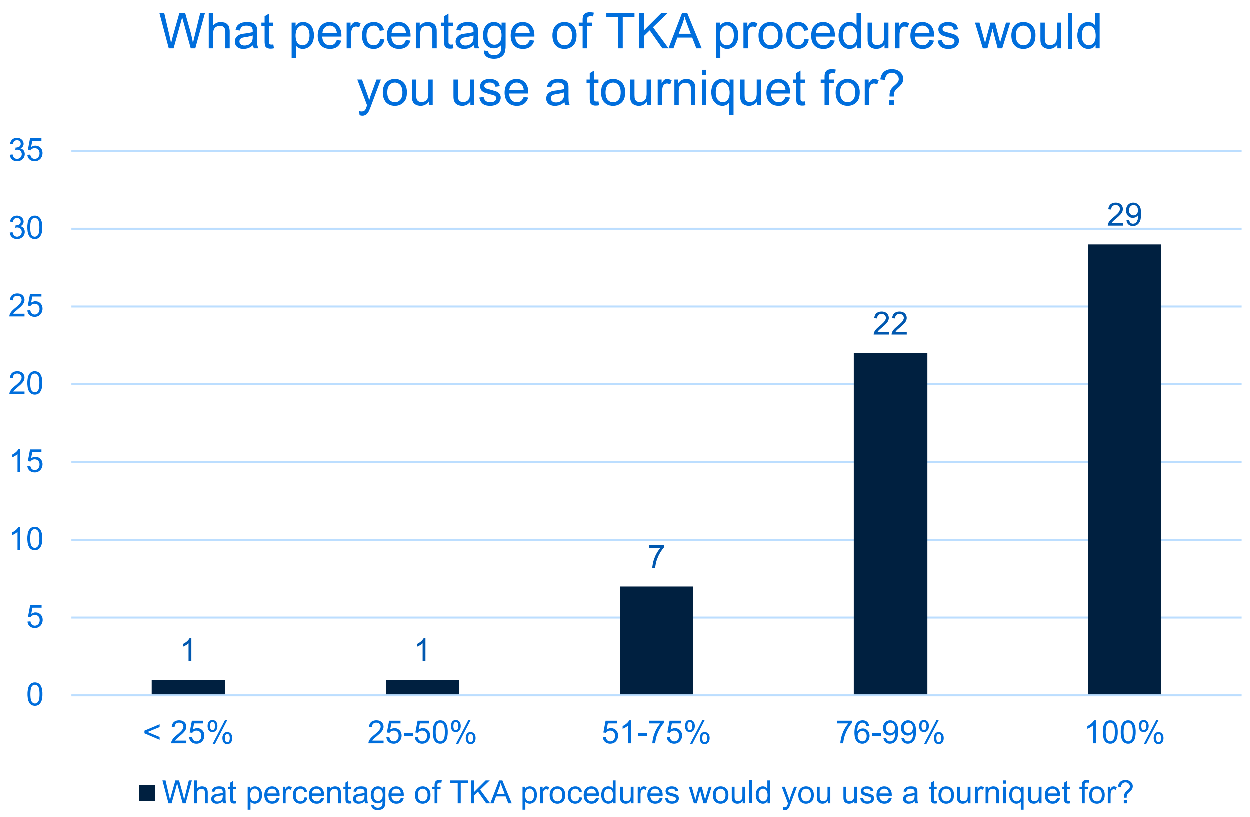

Results – Tourniquet Users

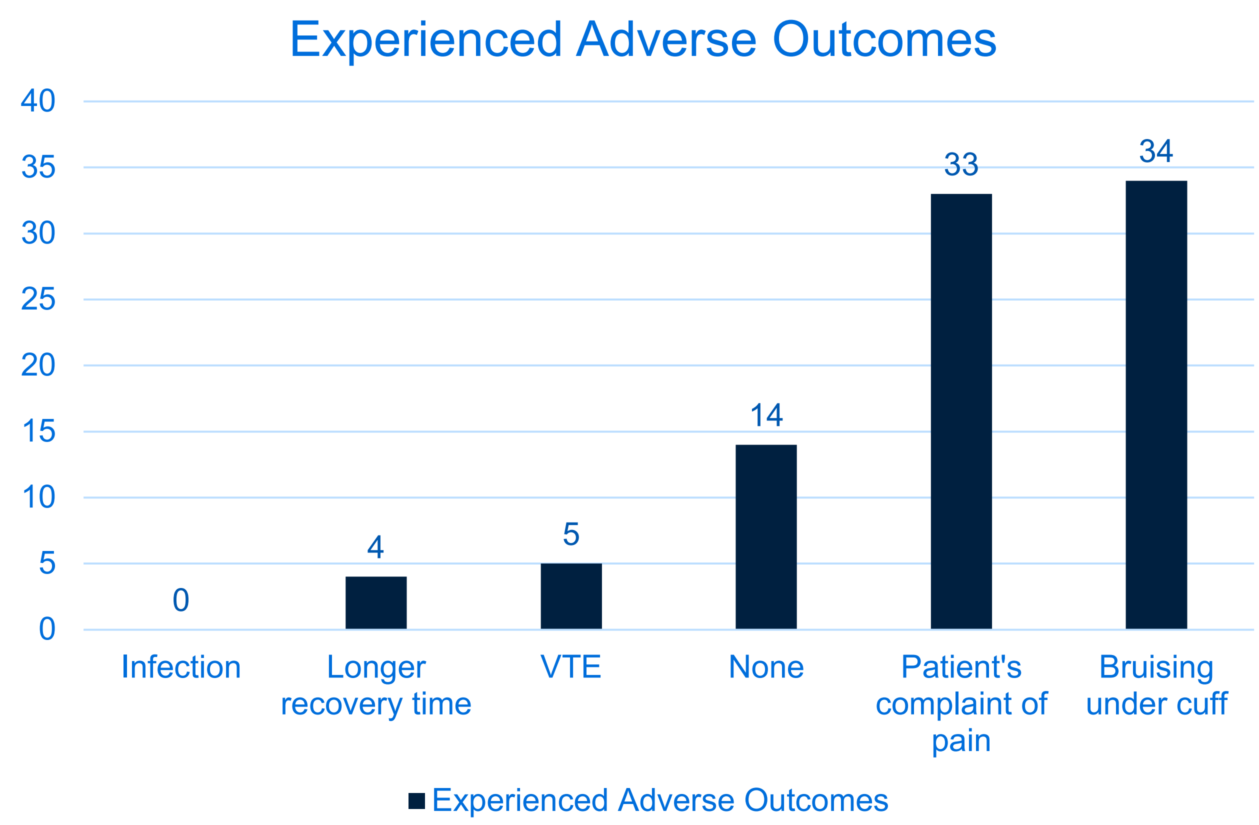

- 23.3 % never experienced adverse outcomes
- 56.7% bruising underneath tourniquet site
- 55% postoperative pain underneath tourniquet site
